# Editorial: Viruses in agricultural systems: Interactions with plants, insect pollinators and fungi

**DOI:** 10.3389/fmicb.2023.1170402

**Published:** 2023-03-23

**Authors:** Islam Hamim, Ken Komatsu, Sean Michael Prager, Beilei Wu

**Affiliations:** ^1^Department of Plant Pathology, Faculty of Agriculture, Bangladesh Agricultural University, Mymensingh, Bangladesh; ^2^Graduate School of Agriculture, Tokyo University of Agriculture and Technology, Fuchu, Tokyo, Japan; ^3^Department of Plant Sciences, College of Agriculture and Bioresources, University of Saskatchewan, Saskatoon, SK, Canada; ^4^Institute of Plant Protection, Chinese Academy of Agricultural Sciences, Beijing, China

**Keywords:** food security, environment, agriculture, viruses, genomics, pathogenesis

Plant pathogenic viruses in agricultural systems significantly reduce crop yields and pose a serious threat to global food security systems. However, the advent of “viromics” in biology has uncovered a plethora of viruses whose effects on ecosystems and food security have not been significantly studied. Studies on viruses are attracting increasing attention in agriculture because of their ability to directly infect crops and the wide-ranging ecological consequences they can have on agricultural ecosystems. Viruses can help plants survive biotic and abiotic stresses under certain conditions. In addition, viruses can interact with bacteria, weeds, pathogenic and symbiotic fungi, insect pollinators, and other species in ways that benefit or harm final crop production.

The Research Topic “*Viruses in agricultural systems: Interactions with plants, insect pollinators, and fungi*” was developed under the sections “Virology” and “Microbe and Virus Interactions with Plants,” which belong to the journal “Frontiers in Microbiology.” The distribution, diversity, transmission, pathogenesis, and control of viruses infecting plants, fungi, and insects in agricultural ecosystems were considered for this topic. However, the final article collection was expanded to include viruses infecting other host species in agroecosystems not listed in the title, considering host-shifting of viruses and phylogenetically related cross-kingdom viruses. This topic has accepted eight original research articles and one review article covering a range of topics related to viruses in agricultural systems. Of these, five of the original research articles focused on the molecular processes of viral infections in different hosts in agricultural systems.

In the work by Gong et al., it was shown that protein lysine acetylation (Kac), an essential post-translational modification in eukaryotes or prokaryotes, has a major impact on the tomato spotted wilt virus (TSWV) infection of *Nicotiana benthamiana*. Gong et al. discovered around three thousand sites linked to acetylated lysine in approximately two thousand proteins by analyzing the acetylation status of proteins in virus-free and TSWV-infected *N. benthamiana* leaves. They also found that after the viral infection, 408 sites on 294 proteins were positively regulated and 284 sites on 219 proteins were negatively regulated. Altogether, 35 conserved motifs were recovered, and K^*^R was the most prevalent combination, accounting for 1,334 (31.63%) of the enriched motifs. According to their bioinformatics study, most proteins with lysine acetylation sites were found in the cytoplasm and chloroplast and were engaged in biological activities such as cellular and metabolic ones.

Chlorophyll a/b-binding protein of the light-harvesting complex II type 1 like (LHC II-1L), which largely controls the integrity of the electron transport system, is an essential component of photosynthesis. Xu et al. investigated the influence of the *Fragaria vesca* LHC II-1L protein (FvLHC II-1L) in strawberry vein banding virus (SVBV) infection. They found that the movement protein SVBV P1 interacted with FvLHC II-1L *in vitro* and *in vivo*. SVBV-infected epidermal cells of *N. benthamiana* leaves displayed co-localization of SVBV P1 and FvLHC II-1L; however, *F. vesca* had elevated FvLHC II-1L protein synthesis. Additionally, Xu et al. discovered that FvLHC II-1L efficiently boosted SVBV P1 to compensate for the intercellular movement of movement-deficient potato virus X (PVXΔP25) and the systemic movement of movement-deficient cucumber mosaic virus (CMVΔMP). Further, it was shown that the overexpression of FvLHC II-1L can enhance SVBV infection of *F. vesca via* interacting with the SVBV P1 protein.

The work by Sun et al. challenged the notion that virions of a virus that infects the host have the same viral genome and properties by examining the genetic variability of the virions of the white spot syndrome virus (WSSV), a DNA virus that affects crustaceans. They found that the WSSV population was made up of a 1:3 ratio of Type A (containing the WSSV lncRNA-24) and Type B (carrying the wsv195 gene), where there are two nucleotide changes between these two types. All virus-infected cells and tissues from different hosts showed a consistent 1:3 structure in viral populations. The study showed that type A WSSV virus infection was boosted by WSSV lncRNA-24 by interacting with WSSV miRNAs in shrimp, but type B WSSV required the wsv195 gene for viral infection. When Type A or Type B WSSV was eliminated from the WSSV population, the copy number of the virus decreased by 100 times in shrimp, and infection was prevented by the simultaneous elimination of both WSSV types. The complementary effects on the WSSV population and the accomplishment of the viral infection by two distinct WSSV types expressing various operational genes show the significance of complementarity between virus population components in viral infection.

Host adaptation causes significant genetic changes in symbiotic microorganisms in insects; however, it is still unknown how virus mutations in symbiotic hosts affect host adaptability to new environments and increase viral fitness in hosts. Acyrthosiphonpisum virus (APV), a symbiotic virus, has undergone genetic divergence in one location, and Lu et al. looked into the impact of this genetic differentiation on the ability of the aphid to respond to unfavorable plants. Single nucleotide polymorphism (SNP) sites in the APV genomes of the pea aphid colonies in *Vicia faba* and *Vicia villosa*, respectively, were discovered using a transcriptome investigation. When host aphids were switched from high-fitness plants *V. faba* to low-fitness plants *V. villosa* or *Medicago sativa*, the SNP at site 5,990, G5990A, located in the RNA-dependent RNA polymerase (RdRp) domain, showed a change from G to A. In RdRp, this SNP resulted in the asparagine (N) to serine (S) substitution at site 196. S196N was located at a random coil distance from the conserved active motifs, and the N196 type of RdRp's polymerase performed 44.5% better than the S196 type. The increased APV replication rate was caused by the enhanced enzymatic activity of RdRp positively impacted insects by lowering plant defenses against aphids, and it sheds light on a scenario in which a host can benefit from modifications of a symbiotic virus when the host adapts to the changed environmental conditions.

*Bemisia tabaci* (Hemiptera: Aleyrodidae) is a highly efficient vector for the spread of the chili leaf curl virus (ChiLCV, Begomovirus), which poses a considerable obstacle to the cultivation of chili in South Asia. Nekkanti et al. investigated the molecular processes driving interconnections between *B. tabaci* and ChiLCV transmission and found potential alternative candidates for the control of the *B. tabaci*-begomovirus complex. Transcriptome analysis of *B. tabaci* revealed 80 genes with distinct expression patterns after 6 h of ChiLCV intake, with 29 being positively regulated and 51 being negatively regulated. The Kyoto Encyclopedia of Genes and Genomes (KEGG) research of the differentially expressed genes (DEGs) revealed their role in metabolic activities, signaling channels, cellular processes, and organismal systems. Reverse transcription quantitative real-time PCR (RT-qPCR) was used to confirm the expression of positively regulated genes following the acquisition of ChiLCV. DEGs that were abundant in *B. tabaci* and promoted viral infection and circulation included replication factor A protein 1, fasciclin 2, inhibin beta chain, cytosolic carboxypeptidase 3, dual-specificity protein phosphatase 10, 15, dynein axonemal heavy chain 17, and Tob1.

*Alternaria alternata* apple pathotype is responsible for Alternaria leaf blotch, a serious fungal disease that reduces apple output worldwide. Interest in mycoviruses as a potential biocontrol agent has grown significantly due to their ability to impart hypovirulence on their hosts. Li et al. found that *A. alternata* f. sp. *mali* strain QY21 was coinfected with *A. alternata* chrysovirus 1 strain QY2 (AaCV1-QY2) and *A. alternata* magoulivirus 1 (AaMV1), which belong to the genera *Betachrysovirus* and *Magoulvirus*, respectively. Hypovirulence of *A. alternata* was associated with these two mycoviruses, with AaCV1-QY2 likely contributing significantly. AaCV1-QY2 can alone postpone development and lessen host virulence, as shown by the fact that the lack of AaMV1 in strain QY21 had no influence on the hypovirulence phenotype. Viruses also prevented strains of *A. alternata* from overproducing the mycotoxin alternariol. AaCV1-QY2/AaMV1 mycoviruses may infect diverse strains of *A. alternata*, which can increase the effectiveness of interspecific dissemination of AaCV1-QY2.

Two original research articles about the diagnosis and characterization of plant viruses are included in this Research Topic. In the first paper, Kumar et al. identified co-infections of tobamoviruses, frangipani mosaic virus (FrMV), and plumeria mosaic virus (PluMV), from the temple tree, or *Plumeria rubra* f. acutifolia. The existence of PluMV was also observed in association with the distinctive symptoms of *Gomphrena globosa* (globe amaranth), a non-host of FrMV. To propagate PluMV, simple rub-inoculation was quite successful, and tobacco, globe amaranth, brinjal, datura, and chili were able to discriminate PluMV from FrMV in a host range assessment. The full genome sequence of PluMV was analyzed, revealing the genomic structure characteristics of tobamoviruses encoding four proteins: small replicase, large replicase, movement protein, and coat protein. The genome of PluMV was longer than that of FrMV; it shared significant sequence similarities with FrMV and other tobamoviruses, and it had a close but diverging evolutionary relationship with FrMV. RT-PCR assays confirmed the natural occurrence of PluMV in temple tree species in India, either alone or in combination with FrMV. In the second research article related to the diagnosis of viruses, Hamim et al. reported that virus-infected leaf samples stored in RNAlater^®^ were acceptable for RT-PCR, PCR, Sanger sequencing, high-throughput sequencing (HTS), and enzyme-linked immunosorbent assay (ELISA)-based diagnostic tests. Agricultural productivity is significantly impacted by viral plant diseases, and accurate detection and characterization of viral infections are crucial for crop disease management. In order to carry out diagnostic tests using sensitive molecular techniques, intact nucleic acids present in plant tissue samples obtained from remote areas are required. RNAlater^®^ offers effective, trustworthy sample preservation by preserving both RNA and DNA in plant tissue samples. Hamim et al. used leaf tissue samples from agricultural fields in Bangladesh that had viral symptoms to assess the viability of this method. Their research suggests that RNAlater^®^ technology could be employed to characterize viruses in samples that have been kept for a long period and obtained from distant areas. This strategy will allow underdeveloped countries with limited laboratory facilities to greatly improve their capacity to identify and control viral infections in agricultural plants using cutting-edge analytical methods.

A comprehensive review article on this Research Topic contributes to our understanding of the biological roles of phosphorylation in the interactions between plants and viruses. One of the most well researched post-translational modifications, phosphorylation regulates multiple cell signaling pathways and is crucial for modulating the viral infection cycle in plants. The molecular mechanisms underlying the phosphorylation of plant virus proteins have been the subject of a considerable number of studies. Zhuang et al. analyzed these findings and classified the impacts on biological processes in accordance with the viral life cycle.

In conclusion, this Research Topic provides state-of-the-art research methods on virus interactions with different hosts in agricultural systems. We would like to take this opportunity to express our gratitude to each of the authors and reviewers, whose outstanding work enabled the publication of this Research Topic. We believe that this collection will increase knowledge, and awareness of the significance of viral diseases, enable better surveillance and potential control of emerging viral diseases in agricultural systems, and prevent epidemics in agricultural crops.

## Author contributions

IH wrote the manuscript. All authors contributed to the article and approved the submitted version.

